# Overreliance on cultural doulas: the paradox of entrusting the communication and care of high-risk migrant women to cultural doulas

**DOI:** 10.1186/s12884-025-07700-2

**Published:** 2025-05-14

**Authors:** Birgitta Essén, Ayanthi Wickramasinghe, Lise Eriksson

**Affiliations:** 1 Department of Women’s and Children’s Health, Faculty of Medicine, Uppsala University, Akademiska sjukhuset, Uppsala, 75185 Sweden; 2 Faculty of Social Sciences, Business and Economics, and Law, Åbo Akademi University, Turku, Finland

**Keywords:** Doula, Maternity care, Migrant women, Midwives, Obstetric care

## Abstract

**Background:**

It is widely recognized that migrant women from low-income countries are considered to be a group with increased obstetric challenges. To address these challenges, cultural doulas were introduced to provide continuous emotional and practical support during childbirth in Sweden. Leveraging their shared cultural background, language skills, and understanding, the idea behind these doulas was supposed to facilitate effective communication between the woman, her partner, and healthcare staff, with the assumption that this would lead to better maternity care for migrants. The aim of this study was to explore healthcare providers reflections on the role of cultural doulas and to explore their perceptions of cultural doulas’ impact on childbirth.

**Methods:**

A qualitative study was conducted in 2022, involving semi-structured interviews with 18 healthcare providers; obstetricians and midwives from two Swedish counties. The data was analyzed using reflexive thematic analysis and discourse analysis, guided by Bacchi’s ‘What Is the Problem Represented to Be?’ approach.

**Results:**

Using Bacchi’s ‘What Is the Problem Represented to Be?’ approach, the analysis highlights how healthcare providers interpreted cultural doulas as an asset in relation to problems in migrants’ maternity care. Three key discourses that emerged were: underlying social and cultural factors, assumptions of improved outcomes and integration, and cultural doulas as informal interpreters. Instead of emphasizing medical risks, healthcare providers focused on social risks and overlooked the importance of professional training.

**Conclusions:**

Cultural doulas are recognized as valuable in addressing gaps in migrant maternity care, yet their role presents a paradox. Entrusting the care of high-risk migrant women to minimally trained non-medical professionals paradoxically risks miscommunication and compromised care quality. Insufficient training, unclear roles, and the overextension of cultural doulas further exacerbate this issue, underscoring the need for systemic reforms. To resolve this paradox and improve maternal outcomes, the maternity care system must redefine the role of cultural doulas, prioritize professional interpretation services, and implement integrated care models tailored to the evidence based medical needs of migrant women.

## Background

Sweden is a country with a highly diverse population, where 21% of residents are foreign-born and an additional 7% of those born in Sweden have two foreign-born parents [1]. Despite Sweden’s healthcare policy aiming to provide equitable care for all [2], the heterogeneity of migrant groups poses significant challenges. Sweden has one of the lowest maternal mortality rates globally, at 4 per 100,000 births [3, 4]. However, since the 1990s, it has been recognized that women from low-income countries (LICs), particularly from sub-Saharan African (SSA) nations such as Somalia, Ethiopia and Eritrea, face the highest rates of maternal and perinatal mortality as well as morbidity, even after migrating to Sweden [5–8]. Studies indicate that migrants from LIC are at elevated risk for severe maternal morbidity such as “near-miss” events [7, 9–13].

Heightened risks during pregnancy, such as a greater prevalence of gestational diabetes and hypertensive disorders like preeclampsia, and perineal injuries have been consistently observed among migrant women from sub-Saharan Africa (SSA) in several European studies, with these conditions being more common among this group of women as compared to others [14–16]. Additionally, this group of women are prone to a spectrum of other conditions, including tuberculosis, rheumatic heart disease, severe anaemia, HIV/AIDS, and malaria, all of which can complicate pregnancy care [10, 17] (Table 1).

For decades it has been known that migrant women from LIC, particularly those from SSA, have elevated risks for adverse perinatal outcomes, including infants who are small for gestational age, post maturity, birth asphyxia and higher rates of perinatal mortality [5, 9, 14]. Suboptimal care factors have been shown to contribute significantly to poorer perinatal health among migrant women. These factors include medical care deficiencies, such as delays in transferring infants with birth asphyxia to neonatal intensive care units, as well as communication barriers due to the lack of authorized interpreters. This long-recognized risk of not using interpreters has been linked to potentially avoidable perinatal deaths [5, 8, 10]. (Table 1).

Women from SSA also experience notable disparities in maternal care. Despite higher rates of breech presentation, they undergo fewer planned cesarean sections, yet have higher rates of emergency cesarean sections, highlighting potential inequities in care provision [6, 14]. Further disparities are noted in the use of epidural analgesia (EDA), with certain migrant groups, such as Somalis, using it less frequently [18, 19]. Additionally, induction rates vary across migrant groups in Sweden, with Somali women being more likely to undergo induction due to post maturity compared to other groups [14, 20]. Similarly, there may be challenges in implementing pregnancy strategies, such as the refusal to accept acute caesarean sections among certain groups of women [21].

In Sweden, there is a task sharing in care of childbearing women, where midwives are the primary caregivers in maternity care, playing a central role in providing comprehensive support that includes psychological, social, and emotional care throughout pregnancy, labor, and postpartum. Midwives are trained to handle most aspects of childbirth and maternal care. Obstetricians, on the other hand, step in for consultations when needed and assume leadership in cases of complications, ensuring that more specialized medical care is provided when necessary.

Migrant women, especially those from diverse backgrounds, often face barriers like language difficulties, cultural misunderstandings, and limited social support, which can impact their maternity care experiences. In this context, culturally responsive care is beneficial, care that is respectful of an individual’s cultural traditions, values, and needs, and addresses their unique circumstances [5, 22].

In order to mitigate these heightened risks faced by migrant women and to provide culturally responsive care, some hospitals in Sweden have implemented cultural doulas, also referred to as community-based doulas [23]. The initiative to introduce cultural doulas in Sweden, supported by governmental funding in 2008, aimed to assist migrant women during childbirth [24]. Despite several Swedish counties having adopted the cultural doula model [25], few critical analyses of the implementation had been done so far.

Cultural doulas are described as a nonrelative support person, who shares a migratory background with the birthing woman and is proficient in her native language as well as the destination country language, who can assist women and their partners in navigating an unfamiliar maternity care system [26, 27]. Cultural doulas were first introduced into Swedish maternity care in 2008 through a community-based initiative led by a non-governmental organization (NGO), supported by governmental funding. Since then, the model has been adopted by various counties, each adapting it to their local context. For example, County A initiated its program in 2016 and County B in 2018. Although cultural doulas are primarily community based, they collaborate closely with hospitals and maternity services. While not formally part of hospital staff, they often accompany women to antenatal visits,during labor and birth, depending on local healthcare arrangements. The structure and coordination of cultural doula services vary across regions, with some municipalities managing them through public health projects or NGOs, often still supported by public funding. Their role is to provide continuous emotional and practical support during childbirth [28]. Therefore, the doula’s language skills and cultural understanding are supposed to facilitate communication between the woman, her partner, and the staff during labor and birth. Cultural doulas, however, do not receive formal training. Instead, they undergo a brief and superficial training lasting between 48 and 64 hours, covering a wide range of topics such as anatomy, physiology, sexually transmitted infections, female genital cutting, pain management, normal and complicated childbirth, Swedish patient laws, breastfeeding, and strategies for continuous labor support [23, 28]. It is important to note that cultural doulas are not recognized as a formal healthcare profession and lack regulation and oversight [27].

A review in 2013 on continuous labor support provided by a doula highlighted several benefits, including fewer low Apgar scores, reduced use of EDA, and fewer instrumental vaginal births, as well as increased rates of spontaneous vaginal births, shorter labor, and improved birth experiences [29], scenarios not reflective of current Swedish obstetric care. Similarly, fewer planned cesarean sections were noted in the review [29], but it is essential to emphasize that reducing cesarean sections is not necessarily positive if it compromises maternal or infant safety. In Sweden, several studies has shown that SSA women do not wish to use EDA [30] Further, the few studies that have tried to evaluate the cultural doulas showed mixed results especially among the most vulnerable migrant group of women from SSA [23, 26, 30, 31]. Women with cultural doulas were less likely to use EDA or baths for pain relief but had higher odds of labor induction and longer hospital stays, with no differences in nitrous oxide use, mode of birth, perineal injuries, or birth asphyxia indicating no effect on severe morbidity outcomes [26]. These discrepancies raise the question of whether the cultural doula concept effectively addresses the specific risks faced by migrant women within obstetric care which have been described above. (Table 1)

**Table 1:** The obstetric outcomes for migrant women from LIC and the effect of support from cultural Doulas/doulas respectively

	Obstetric outcomes for migrant women from LIC	Effect of support from cultural doula/doulas
**Maternal Health**	Increased risk of: Gestational diabetes Preeclampsia Severe maternal morbidity Maternal mortality	**No impact**
**Perinatal Health**	Increased risk of: SGA/IUGR Asphyxia Perinatal mortality	**No impact**
**Obstetric interventions**	Delayed neonatal intensive care Increased risk of emergency CS Low use of EDA Suboptimal care Perineal injuries Increased odds for induction due to post maturity	Decreased use of EDA and bath Increased odds of induction Decreased instrumental vaginal births

The emergence of the cultural doulas is relatively new with limited research on the subject. Some qualitative studies have been conducted with migrant women and the cultural doulas [32–35] but few with healthcare providers (HCPs) [27, 32, 36]. Therefore, this topic warrants critical and exploratory study due to the limited research and the gap between obstetric risks and the potential impact of cultural doulas. This study seeks to provide insights into the role of cultural doulas from the perspective of midwives and obstetricians. The challenges related to the distribution of roles and responsibilities among HCPs, cultural doulas, and authorized interpreters, as well as how HCPs identify and perceive the issues and their proposed solutions, will be explored. The aim is to explore HCPs reflections on the role of cultural doulas and to explore their perceptions of cultural doulas’ impact on childbirth. The research questions are:

1. How do HCPs reflect on the social and medical problems and risks in migrant women’s maternity care and on the effects of the cultural doula concept with regards to their (HCPs) knowledge about migrant women’s adverse obstetric outcomes?
2. How do HCPs reflect on the role of the cultural doula in contrast to the professional duties of midwives and authorized interpreters, respectively?

### Theoretical framework

We use Bacchi’s (2016) WPR approach (What’s the Problem Represented to be?) to analyse how HCPs represent sociocultural and obstetric risks and suboptimal care in migrant maternity care, and challenges in the division of professional roles. Additionally, we analyse how the cultural doula support is understood as a solution to these problems. Bacchi’s approach is relevant for this study as a discourse analytical framework for analyses of policy-driven solutions to societal problems or health policy problems. It is based on a set of analysis questions, exploring how problems are represented, their underlying assumptions, what has not been problematized, and effects of how these problems are represented. Hence, the approach focuses on how problems are discursively constituted and given meaning.

## Methods

### Study design

This study was a part of a larger qualitative study [27] using semi-structured interviews. An interview guide was developed based on previous research and clinical experience. The interview guide comprised of four broad topics that were relevant to the research questions such as, background and definition of the problem, labour support in Sweden, and interprofessional dynamics.

### Setting

This study examines the cultural doula concept in two counties in Sweden. In both counties, migrant women receive doula support through maternity clinics. In County A, cultural doulas provide support before and after labor, whereas in County B, doulas also offer support during labor. Although the doulas countries of origin are not specified in either county, the languages available are listed. County A primarily focuses on offering cultural doulas who speak Arabic, Tigrinya, Somali, and Dari. County B includes these languages and additionally offers support in Persian, Mongolian, Russian, Spanish, Turkish, Ukrainian, and Urdu.

### Participants

A total of 18 HCPs were selected for this study, comprising 3 obstetricians and 15 midwives. HCPs were recruited from different hospitals and primary care centers across two counties, providing a diverse perspective. The participants were chosen based on their extensive experience working with cultural doulas, whether in clinical, organizational, or administrative capacities. These participants were chosen as their insights could provide valuable perspectives on the integration and impact of cultural doulas within the maternity care framework. Some key informants were also interviewed who were relevant and could provide further insight into the cultural doulas. In addition to the above a scoping analysis of policy text was also carried out.

### Reflexivity

The authors approach the topic through a research program focusing on social values, migration, and equity in sexual and reproductive healthcare. The first author is Professor of international maternal and reproductive health, committed to developing comprehensive reproductive health services to women in unprivileged situations. The second author is a researcher in global health with background in medicine, and the third author is a sociologist involved in interdisciplinary research on reproduction, childbirth and social norms.

### Data collection

A purposeful sampling strategy was employed and complemented by a snowball approach. Interviews were conducted both online and face-to-face from January to April 2022, with each interview lasting approximately 30 to 90 min. The interviews were conducted in Swedish by the research team. All interviews were audio-recorded with the respondents consent, and participants were given the option to choose between on-site or online interviews.

### Data analysis

The data analysis was carried out using a two-stage qualitative approach. First, we applied reflexive thematic analysis, inspired by Braun and Clarke’s [37] six-step framework, by coding the material using both latent and manifest codes to identify emerging themes and patterns relevant to our research questions. Second, to further examine underlying narratives, we used Carol Bacchi’s “What’s the Problem Represented to be?” (WPR) [38] approach to guide our discourse analysis.

The data were initially transcribed verbatim in Swedish, then translated and back-translated into English to ensure reliability. Bacchi’s WPR questions were used to structure the results and highlight tensions between respondents’ knowledge of obstetric risks and their perceptions of the role of doula support. The entire research team was actively involved in analyzing the data, ensuring intercoder reliability through regular collaborative discussions and by reviewing each other’s coded material [39]. For clarity and readability, quotes translated from Swedish were stylized in English.

## Results

Applying Bacchi’s WPR approach, the analysis reveals how problems in migrants’ maternity care are constructed and understood by HCPs. Three key discourses emerged: underlying social and cultural factors, assumptions of improved outcomes and integration, cultural doulas as informal interpreters.

### Underlying social and cultural factors

Despite evidence that migrant women from LICs have a higher obstetric risk, the participants did not highlight these medical issues but instead focused on other underlying factors such as cultural disparities and socioeconomic vulnerability. Maria articulates this perspective:

“We have evidence of the vulnerability, both physical or medical and psychological and social and all the pieces a pregnancy entails. And not being… being either newly arrived or not integrated, not knowing the language, it leads to so much increased risks and adverse outcomes that you need a holistic perspective on it, and in that the cultural interpreter doulas have added a lot to us other professions that assist women and families.” (Maria, midwife).

Maria highlights that the medical risks for migrant women are compounded by sociocultural disparities, such as lack of integration and not speaking Swedish, views that were echoed by other HCPs as well. However, despite the well-documented, more serious adverse obstetric outcomes and enhanced risks for specific migrant subgroups, these are not discussed in detail by the HCPs. Instead, the focus remains largely on communication barriers. Even when some HCPs, like Magda, acknowledge the increased medical risks for migrants from certain regions, such as SSA, the primary concern continues to be communication challenges:

“We know that there are certain medical conditions in obstetrics with heightened risk factors for those from countries south of Sudan or in sub-Saharan Africa. Additionally, there are medical risks associated with difficulties in communicating symptoms.” (Magda, physician).

Several participants identified communication barriers as a major issue impacting the quality of care for migrant women, similar to Magda’s experience. Anna illustrates how misunderstandings can obscure obstetric risks:

“Communication can be challenging regardless of whether you come from a hut or a castle. We have seen that background sometimes doesn’t matter, because you still encounter difficulties and misunderstand each other, so that we may miss identifying the risks before pregnancy and childbirth.” (Anna, midwife))

Some HCPs assumption of low literacy among migrant women also shapes the care approach, potentially overlooking the need for tailored communication strategies:

“We have some women who cannot read and write in their mother tongue and are illiterate…how can they remember everything we say? They cannot write down, not document, they can never get written information from us that they understand. Which becomes very difficult for them.” (Lina, midwife).

The responders addressed how migrating from different health systems can lead women to have different expectations from the healthcare system of the receiving country. These varying expectations often result in misunderstandings, and mistrust among HCPs and migrant women leading to poor obstetric outcomes. While some respondents described this as a culture clash, others viewed it as a broader issue not specifically tied to cultural differences. Selma noted that these differing expectations could lead to misunderstandings, causing migrant women to feel unsupported:

“[…] it can also stem from traditional, cultural, or experience-based misunderstandings. This may involve differing beliefs about what is appropriate for a pregnant woman, past negative experiences with medical care in their home country, or experiences during their journey to Sweden. Additionally, the treatment by medical staff in Sweden—such as long wait times—can create suspicion towards caregivers. Consequently, the support and security we try to provide may not effectively reach them.” (Selma, midwife).

Some participants emphasized that they had experiences where migrant women held specific expectations related to their experiences in their country of origin. These expectations can be broad and often diverse. For instance, some HCPs reported that certain migrant groups view CS as the preferred delivery method due to its association with higher social status, while others have a profound fear of CS due to negative connotations in their home countries. Johanna illustrated this fear, saying:

“[Migrant] women, the only thing they want is that we note in the statement is that ‘definitely does not want to undergo caesarean section’, because that means death in their home nation.” (Johanna, midwife).

This suggests that beliefs about childbirth may play a significant role in shaping migrant women’s expectations and experiences in Sweden. Additionally, HCPs emphasized the need to address these views, which they saw as cultural, although they were influenced by the classic socioeconomic limitations of healthcare systems in the migrants’ home low-resource countries and not related to the culture. Not understanding Swedish protocols can also lead to noncompliance, fostering feelings of substandard care and discrimination. Eva pointed out another significant difference:

“[…] another one of those typical culture clashes is that in Sweden it is the midwife that supervises uncomplicated pregnancies and in many other countries it is the doctor and that can create a lot of insecurity for the woman. ‘why don’t I get to see a doctor, is it because I’m an immigrant? Do I receive worse care than Swedish women because I only get to see a midwife?’” (Eva, midwife).

One of the participants shared her perspective on how past experiences can shape fears around childbirth. She explained that in some places, midwives are viewed with suspicion, sometimes even seen as harsh figures, leading women to fear punishment for expressing pain during labor:

“We also talked about it with one of the doulas, that in some countries they say that midwives are witches…. that they can fight during delivery, the woman must not scream, or then they will get hit, for example, when giving birth.” (Martina, midwife).

The participants misidentified problem areas mainly as cultural differences, as opposed to differences in the health systems and past experiences. Therefore, the HCPs focused more on social causes than medical risks when describing problem areas, which is important to keep in mind when understanding their expectations for the cultural doula’s role.

### Assumptions of improved outcomes and integration

The respondents’ representations of problems and risks are built on an underlying assumption that the involvement of cultural doulas led to improved outcomes by bridging communication gaps and providing support, which enhances patient safety and reduces medical risks. Maria reflects on this perceived benefit:

“[Cultural doulas have] added a great deal of improved quality and improved outcomes, I would say. It’s all about being the contact so that you can manage to call the labor ward, which can minimize many risks, to promote safety—we know safety prevents a lot of things—including medical outcomes. They are there and can also guarantee in the best way that I’m understood, that I faster can help and assist and prevent things.” (Maria, midwife).

A key requirement for a cultural doula is the ability to speak the same language as the migrant woman. It underscores the impact the doula is seen to have enabling migrant women to communicate directly with someone in their own language. Lina assumed that the information may be easier to take in this way.

“I give a lot of information, but it is usually only once and there is quite a lot of information I give about many different things, so sometimes you have to digest it and maybe also get it in your mother tongue […] We have, use an interpreter but that is also like a second-hand information and maybe it needs, it is easier to get it through a person you can talk to directly and see many times.” (Lina, midwife).

Some HCPs perceived that cultural doulas play a vital role in bridging the gap between migrant women’s expectations and the Swedish healthcare system. They assume that doulas help clarify information and provide reassurance, which can alleviate some of the confusion and mistrust faced by migrant women. HCPs described positive relationships between migrant women and cultural doulas, highlighting the doula’s innate understanding of the women’s needs due to shared backgrounds. As Katarina expressed:

“One must have someone who acts as a bridge between these two cultures: our Western medical care, where we often believe we know best, and the perspective of the migrant woman. The cultural doula possesses a different understanding than I do; she has insights into the woman’s life experiences that I do not have access to.” (Katarina, midwife).

Lova, for instance, highlights the importance of cultural doulas’ own lived experiences, assuming their familiarity with integration into Swedish society and Swedish values:

“I think it’s great that the culture doulas exist but I also feel like they have too little time. These are women who themselves have been integrated into Swedish society and know what it entails. That person has both sides of the coin in a way.” (Lova, midwife).

Similarly, Anna’s perceptions of cultural doulas as playing a transformative role, likening them to a bridge that supports migrant women’s journey into Swedish society:

“[Doulas] are an ambassador for the woman, not just giving them a small span to cross into our culture but a giant bridge that guides them forward.” (Anna, midwife).

The role of cultural doulas is often seen as pivotal in enhancing the experience and outcomes for migrant women in healthcare settings, particularly by aiding their integration and promoting a sense of belonging. HCPs perceived cultural doulas as a ‘universal fixer’s’, not only as facilitators of language and communication but as bridges to understanding and navigating Swedish culture and healthcare practices.

### Cultural doulas as informal interpreters

HCPs in administrative positions acknowledged a lack of awareness about the limitations of the cultural doula’s role. However, the insufficient training for cultural doulas was largely overlooked by HCPs, which raises concerns about their ability to handle complex medical situations. Respondents noted that, although cultural doulas were not meant to provide medical advice or serve as interpreters, they often found themselves in these roles due to challenges in obtaining authorized interpreters, particularly in county B. However, the cultural doulas in county A were not present at labor, and mostly met the women privately. Several respondents in county B described cultural doulas as a better alternative to authorized interpreters due to doulas being more present, available, supportive, and having more knowledge about childbirth.

“The cultural interpreter doula replaces the interpreter. […] So that there is like an interpreter and a doula in one. […] If I had to choose, I would always choose a cultural interpreter doula first precisely because I get that security and support and someone who mediates the process; she becomes a support to the midwife also in conveying security and what happens next.” (Julia, physician).

Magda found that cultural doulas supported the conversations with women in a different way than interpreters:

“I think that sometimes they can kind of deepen what I’m saying. And perhaps clarify in a different way than an interpreter does.” (Magda, physician).

Katarina mentioned that cultural doulas interpret as sisters:

“She is used as an interpreter, and she should ideally not do that. […] Because it depends on what it’s about, she must interpret as a sister. And this is a bit difficult because I never have an authorized interpreter plus a cultural interpreter. […] But she doesn’t translate what I say verbatim.” (Katarina, midwife).

This overlap created confusion between the functions of doulas and authorized interpreters, resulting in concerns about the accuracy and appropriateness of the information conveyed. Eva highlighted the potential risks associated with doulas acting as interpreters:

“I feel that there is a risk that the healthcare staff uses the doula as an interpreter, even though they know that they are not trained as interpreters. […] The doula is well aware that in a situation where she does not feel that she can transmit information, she has to speak up very sharply, but just the fact that the healthcare staff risks considering her an interpreter is a risk.” (Eva, midwife).

The reliance on cultural doulas as de facto interpreters assumes that they can sufficiently fill this role despite lacking the formal training of authorized interpreters. Maria implicitly supports this view:

“They also interpret the language, so they do a translation, which we have discussed a lot based on the fact that they are not authorized interpreters, which in itself is a profession that requires 3 years of training and guidance and development and everything, but they are ‘as good as it gets’.” (Maria, midwife).

Further emphasizing the gaps in the current system, Lova remarked:

“It’s not medical; we don’t assign any medical knowledge to a culture doula. It’s about culture.” (Lova, midwife).

In general, the participants felt that while cultural doulas serve as valuable intermediaries in the maternity care system, they are ultimately an imperfect solution to a complex problem. Participants recognized that cultural doulas can provide crucial support and bridge gaps between cultures, yet acknowledged that they cannot fully resolve the underlying issues that contribute to these challenges.

A cultural doula is not supposed to act as an interpreter, yet respondents identified them as unofficial interpreters. Another paradox was that even though identifying communication as a major problem area, HCPs chose to use the cultural doula as an interpreter. The use of cultural doulas as substitutes for authorized interpreters is seen as a practical solution to communication barriers but introduces new risks, such as miscommunication or misinformation. Maria points out the logistical challenges:

“[…] we can’t have an interpreter standing… 20 hours in the same delivery room for example […] and this is what we have been discussing for so long, that the birthing mothers have not had their rights fulfilled to be interpreted and get an interpretation, because the administration have had such difficulties finding solutions to this.” (Maria, midwife).

This substitution is based on the assumption that having multiple people, such as cultural doulas and interpreters, in the room could complicate rather than simplify communication. As a result, many respondents indicated that they typically do not have both a cultural doula and an authorized interpreter present, implying that the doula often takes the place of the interpreter. Stina explained the practical implications of this arrangement:

“I have never had both a cultural interpreter doula and an authorized interpreter. It would probably have felt a bit excessive if I am to be honest.…on one end you have the partner and then you have a cultural doula and an interpreter (who may be by phone). I think it can become very unclear and then it is difficult to know…It might work the feeling is just that with so many people maybe the cultural doula would clarify what the interpreter says…it’s like a game of telephone and then you do not know who said what. So, I think the feeling would have been a bit much but like I said I have no experience of it.” (Stina, midwife).

Overall, while the participants stated that cultural doulas played a crucial role in improving communication and providing support, their use as informal interpreters and the focus on cultural vulnerabilities over specific obstetric risks highlight significant contradictions.

## Discussion

This study highlights significant concerns and contradictions in the implementation of cultural doulas within Swedish maternity care, particularly concerning the medical and social care of migrant women with increased obstetric risks. By applying Bacchi’s WPR framework, we analyze how the widely recognized challenge of adverse obstetric outcomes among migrant women is represented and how solutions, such as the introduction of cultural doulas, are discursively framed. Cultural doulas were introduced with the intent to bridge communication and cultural gaps, yet this research shows that their role is often misunderstood and extended beyond its intended scope, revealing systemic challenges that may unintentionally heighten risks for migrant women.

The WPR framework reveals that, when discussing problem areas, HCPs tended to focus on underlying factors such as communication and culture, rather than directly addressing the risks associated with specific obstetric outcomes. Although some HCPs acknowledged the well-known fact that migrant women from low-income countries (LIC) may face more serious health consequences, they often did not refer to specific obstetric outcomes, which suggests a possible lack of awareness or knowledge about these risks [40]. The HCPs identified key issues as related to communication barriers, diverse cultural backgrounds, and socioeconomic vulnerabilities. Some responses from HCPs indicated a sense of mistrust from migrant women toward HCPs. However, this contrasts with findings from a study on antenatal care for SSA migrants in Sweden, where participants reported high levels of satisfaction with their HCPs and expressed diminished need for additional emotional support [30]. This suggests a disconnect between the perceptions of HCPs and the actual experiences of migrant women, highlighting the need for a more comprehensive understanding of their healthcare needs.

HCPs in the study identified communication as a major issue, a finding consistent with previous research [10, 13, 41–43]. However, the cultural doula’s job description states she should not substitute, an authorized interpreter. Especially due to the fact that women in Sweden that do not speak Swedish have the right to get an interpreter during labor and birth (Swedish Healthcare Guide 2024). Furthermore, Swedish policies and clinical guidelines recommend the use of authorized interpreters to address communication barriers [44, 45]. However, our findings reveal significant paradoxes. Despite recognizing communication as a challenge, cultural doulas were perceived by the participants as a solution without thorough risk assessment, despite limitations in their role and being unskilled in this area. As a result, communication barriers may remain unaddressed by HCPs, potentially endangering both mother and child. For instance, perinatal mortality, a devastating consequence of failing to use an authorized interpreter, may persist if professional interpreter services are not consistently employed [5]. Some studies have also shown that the cultural doulas themselves have language difficulties in understanding the HCPs [46]. Only a few respondents in our study mentioned the potential risks of using cultural doulas for interpretation, a concern also echoed in previous research. Unauthorized interpreters have been shown to reduce the quality of care received by migrants [43, 45, 47], and the risks posed by untrained interpreters such as doulas, due to their lack of formal education and potential lack of impartiality, have been well-documented in prior studies [48–50].

In our study, respondents perceived the cultural doula’s role as more suitable for the interpretation needs in labor wards. They emphasized the doula’s continuous presence throughout labor, her more personal connection with the patient, and her perceived familiarity with obstetric terminology gained from experience with multiple labors. However, there was minimal awareness regarding the doula’s absence of training in interpretation and lack of medical knowledge. A report by the National Board of Health and Welfare previously identified clinical setting challenges and availability of authorized interpreters as reasons why HCPs sometimes turn to untrained interpreters, like cultural doulas [47, 51, 52]. Some HCPs also expressed concern that this practice could lead to critical misunderstandings, particularly when cultural doulas are expected to convey complex medical information that exceeds their training [26]. Therefore, the assumption by HCPs that a cultural doula can adequately replace an authorized interpreter—especially when migrant women face significantly more communication challenges with potentially serious consequences—creates a troubling paradox, resulting in even more suboptimal care, the opposite of the intended outcome.

This overreliance on cultural doulas highlights a broader resource constraint within the healthcare system, where professional interpretation services and HCPs—essential for the care of migrant women—are often unavailable, forcing cultural doulas to fill a gap they cannot fill leading to a quality trap [36]. In some instances, participants appeared willing to delegate not only communication tasks but also direct aspects of professional patient care to cultural doulas. This included nursing responsibilities and tasks traditionally performed by midwives, such as monitoring maternal well-being, supporting the labor process, and providing psychological and emotional support, particularly when staff were short-handed or when they constrained by time [25, 36]. Contrary to our study, however, some studies have shown that HCPs felt doulas were duplicating or taking away their duties [48, 49, 53], a sentiment not expressed by our participants. This delegation or “quick fix” approach, risks undermining the quality of medical care provided. It also detracts from the cultural doula’s intended role, potentially leading to a lack of clarity in care responsibilities and a dilution of medical effectiveness. Relying on an imperfect solution to address complex needs only further reduces the quality of care for the most vulnerable group of women.

According to the WPR lens, a key issue frequently highlighted by HCPs was ‘cultural issues’, though none of them provided clear explanations of what these so-called ‘cultural issues’ entailed. HCPs often drew a sharp distinction between medical and cultural knowledge, positioning cultural doulas as intermediaries tasked with offering cultural support while overlooking the complex medical needs of migrant women [6, 7, 54]. Notably, there is no evidence to suggest that pure cultural factors contribute to adverse birth outcomes, nor have documented instances of cultural clashes in delivery rooms been reported [8, 54]. Challenges arise primarily when migrant women from SSA are expected to attend antenatal care visits, which they may perceive as unnecessary due to the absence of illness—a perspective shaped by their socioeconomic context rather than cultural beliefs [21]. As mentioned in the introduction, women who had cultural doulas were less likely to use EDA, and were more likely to have induced labor, longer hospital stays and lower rates of CS [26] (Table 1). Additionally, the presence of cultural doulas has been linked to less favorable aspects of the maternity care experience, suggesting potential drawbacks in their role [27]. This lack of knowledge and understanding can perpetuate a fragmented care model that inadequately addresses the medical needs of migrant women, potentially leading to harmful miscommunications [5, 10]. Furthermore, the lack of awareness regarding how to effectively implement research findings seems to be driven by emotions, cultural assumptions, and ideology rather than by evidence-based knowledge.

The findings highlight HCPs perceptions and expectations of cultural doulas performing clinical tasks such as interpreting, conflict with the doulas’ intended non-clinical roles and responsibilities. Their training, typically limited to 48 to 64 hours [23], is designed to prepare them for providing emotional and cultural support rather than interpreting complex medical procedures. Yet, HCPs frequently expect cultural doulas to perform beyond their training, either as informal interpreters or as advisors on medical matters as well as acting as a substitute for midwives when they are overburdened. This role confusion not only places doulas in challenging situations but also compromises patient safety and care quality. As some participants noted, relying on doulas as “good enough” substitutes for authorized interpreters undermines the integrity of the healthcare system and reflects a broader systemic issue that goes beyond individual doula training [27, 35].

Migrant women were described by HCPs as facing obstacles related to their limited knowledge of the Swedish healthcare system, including having different expectations of care, difficulty navigating the system, and lower health literacy. There was an assumption among some HCPs in the study that low literacy in migrant women also correlated to a lack of understanding and comprehension. Other HCPs described cultural doulas as purely playing a key role in helping migrant women understand the Swedish healthcare system, offering support in bridging these gaps, providing information, and acting as a guide, which is the role of a midwife. There was diverging descriptions of the information provided by the cultural doula, which includes discussing pregnancy-related topics. Some responders said the information should be experience-based, whereas others said fact-based. This presents another contradiction based on the cultural doula’s limitations of not giving medical advice. There were multiple instances where medical competence was implied for the cultural doula to carry out her assignment. If this information should be experience-based, it creates risks of misinformation, whereas if arguing that information about this should be fact-based, it supports the argument that the educational training is not enough, which has been discussed in a study with cultural doulas [36]. The same study also reported incidents where the cultural doula found themselves to interpret symptoms and act as a mediator between the migrant woman and the HCPs [36]. This point was reinforced by the cultural doulas themselves admitting that they felt their education was insufficient to effectively fulfill their role [35]. This further supports the contradictions regarding the cultural doula’s role and remaining within her limitations. Another aspect that makes it more difficult for the cultural doula to stay within limitations is the described needs of the migrant woman. HCPs in our study explained that there was an increased need for information for migrant women and that there were not enough resources, primarily time, to address these. This suggests that resource shortage leads to outsourcing midwives’ traditional duties to provide adequate information and answer questions these women might have, to untrained cultural doulas.

The study highlights a significant conflict between the goals of cultural integration and the medical risk management necessary for migrant women’s maternity care. The cultural doula initiative is often framed as an integration tool, aimed at enhancing cultural understanding and communication between migrant women and HCPs [28]. This perspective assumes that improved cultural competence will naturally result in better health outcomes. However, while cultural sensitivity is important, it may obscure the specific medical needs and risks faced by some migrant groups [27, 54], particularly women from LIC such as SSA countries where the practice of using doulas doesn’t exist. This singular focus on cultural support may simplify the challenges these women face, risking a misalignment with their medical needs and delaying necessary interventions. The greatest paradox lies in delegating HCPs responsibilities and using cultural doulas as interpreters, leaving the most vulnerable migrant women in the care of cultural doulas who have only a few hours of training.

### Strength & limitations

This study has several strengths. It included HCPs engaged with cultural doulas in various contexts, allowing for a comprehensive depiction of the concept through their shared reflections. Participants were recruited from different hospitals and primary care centers across two counties, providing diverse perspectives.

However, some limitations should be noted. The term “migrant” is broad and does not capture contextual nuances, resulting in a heterogeneous group. While the researchers sought clarifications during interviews, the data did not allow for subgroup analysis, which future studies could explore. Additionally, since cultural doulas are still in the project phase, responses—particularly from administrators—may be biased toward securing continued funding. However, the consistency of views across different HCPs helps mitigate this concern.

Finally, while the study captures HCPs’ reflections on cultural doulas, it cannot fully account for the interactions between cultural doulas and migrant women, as these often occur outside care settings or in languages the HCPs do not understand. Input from organizational HCPs, who are closely involved in these projects, provided valuable context to address this gap.

## Conclusion

Migrant women, particularly those from LICs, are at higher risk of adverse obstetric and neonatal outcomes. Given these heightened risks, it is essential that their care is supported by professional interpreters and HCPs. However, a troubling paradox arises when these women, who require specialized care, are instead given cultural doulas—non-medical professionals with minimal training—often in roles that go beyond their capabilities, such as informal interpreters, advisors, or caregivers. This overreliance on cultural doulas can lead to miscommunication and compromised quality and safety in obstetric care.

Moreover, while cultural doulas can enhance communication and understanding, they cannot replace professional interpreters or HCPs in delivering high-quality care. This study highlights a significant issue within Swedish maternity care: the unclear roles of cultural doulas, which contribute to their misapplication in roles such as communication and the delivery of clinical information—tasks that should fall to midwives and HCPs. The confusion about their roles, combined with insufficient training, leads to challenges in meeting the complex medical needs of migrant women. Rather than being an issue with cultural doulas themselves, this reflects a broader challenge within the maternity care system, where there is a lack of clarity regarding the division of responsibilities. Despite these challenges, midwives are increasingly relying on cultural doulas to assist with tasks like providing medical information and support. This shift does not lead to improved maternal or neonatal outcomes and may unintentionally perpetuate disparities in care.

These paradoxes reflect broader systemic inequities in maternity care for migrant women, including fragmented care and a lack of resources. Addressing these issues requires a reevaluation of the role of cultural doulas, ensuring they are properly trained, and prioritizing professional interpretation services alongside culturally competent care. By fostering a more balanced and integrated model, Sweden’s maternity care system can better meet the complex needs of migrant women, ultimately improving maternal health outcomes and safeguarding the care of this vulnerable population.

Given the vulnerabilities of migrants from LICs, it is crucial for Sweden’s maternity health system to provide care that directly addresses their needs and is evidence-based. This can be achieved by ensuring that HCPs have clear roles and responsibilities, particularly regarding the use of cultural doulas. Cultural doulas play a valuable role in supporting migrant women, but it is important that their duties are clearly defined to avoid confusion with other roles, such as interpreters, and to ensure that qualified professionals handle clinical aspects of care.

## Data Availability

The data analysed in this study is available from the corresponding author on request.
